# Social inequalities in changes in health-related behaviour among Slovak adolescents aged between 15 and 19: A longitudinal study

**DOI:** 10.1186/1471-2458-8-57

**Published:** 2008-02-12

**Authors:** Ferdinand Salonna, Jitse P van Dijk, Andrea Madarasova Geckova, Maria Sleskova, Johan W Groothoff, Sijmen A Reijneveld

**Affiliations:** 1 Department of Educational Psychology and Health Psychology, Faculty of Arts, P.J. Safarik University, Moyzesova 16, 040 01 Kosice, Slovakia; 2 Department of Health Sciences, University Medical Center Groningen, University of Groningen, Antonius Deusinglaan 1, 9713 AV Groningen, The Netherlands

## Abstract

**Background:**

Lower socioeconomic position is generally associated with higher rates of smoking and alcohol consumption and lower levels of physical activity. Health-related behaviour is usually established during late childhood and adolescence. The aim of this study is to explore changes in health-related behaviour in a cohort of adolescents aged between 15 and 19, overall and by socioeconomic position.

**Methods:**

The sample consisted of 844 first-year students (42.8% males, baseline in 1998 – mean age 14.9, follow-up in 2002 – mean age 18.8) from 31 secondary schools located in Kosice, Slovakia. This study focuses on changes in adolescents' smoking, alcohol use, experience with marijuana and lack of physical exercise with regard to their socioeconomic position. Four indicators of socioeconomic position were used – adolescents' current education level and employment status, and the highest education level and highest occupational status of their parents. We first made cross tabulations of HRB with these four indicators, using McNemar's test to assess differences. Next, we used logistic regression to assess adjusted associations, using likelihood ratio tests to assess statistical significance.

**Results:**

Statistically significant increases were found in all health-related behaviours. Among males, the most obvious socioeconomic gradient was found in smoking, both at age 15 and at 19. Variations in socioeconomic differences in health-related behaviour were more apparent among females. Although at age 15, almost no socioeconomic differences in health-related behaviour were found, at age 19 differences were found for almost all socioeconomic indicators. Among males, only traditional socioeconomic gradients were found (the lower the socioeconomic position, the higher the prevalence of potentially harmful health-related behaviour), while among females reverse socioeconomic gradients were also found.

**Conclusion:**

We confirmed an increase in unhealthy health-related behaviour during adolescence. This increase was related to socioeconomic position, and was more apparent in females.

## Background

Whereas adolescence is traditionally viewed as an age period of good somatic health, psychosocial health variables, e.g., psychosomatic health complaints or health-related behaviour (HRB), play a decisive role in determining adolescents' self-perceived health.

Over the last thirty years a number of studies describing the relationships between socioeconomic position (SEP), health-related behaviour HRB and health have been performed [1-7]. Undoubtedly HRB is a very important determinant of health as well as a contributor to socioeconomic inequalities in health [2,8].

Since adolescents' psychosocial health problems may have major implications for adult morbidity and mortality, investigating their correlates, such HRB deserves priority. With respect to HRB, adolescence is one of the most important periods of life. Adolescence is characterised by a strong tendency to experiment with risk behaviour. The desire for novelty and the courage for experiment are much greater in adolescence than in later life [9]. Despite it being illegal, many young people have experience with drinking alcohol before turning 18, likewise with using drugs such as marijuana [9,10]. Most adult smokers took up regular smoking in the period of adolescence [11,12]. Even if most students are physically active at school, their compulsory school involvement often fails to translate itself into leisure time physical activity later [13,14]. Moreover, the influence of peers and youth subcultures on HRB is statistically significant [15,16]. HRB established during this period tends to be maintained into adulthood [17-19]. Due to the characteristics mentioned above, adolescence is also a very sensitive period for interventions and policies aimed at promoting health by focusing on risk behaviour [20].

Previous research has consistently documented social class gradients in child and adult health [21]. Low SEP adults are more likely to engage in risky health behaviours [22].

Findings among adolescents are not so consistent. Previous research has shown a very strong traditional (consistent with adult behaviours) socio-economic gradients in insufficient physical activity of adolescents [6,14,23-25]. Also regarding smoking by adolescents, mostly traditional socio-economic gradients were found [21,26], though there are few studies which reporting no [6,27,28], or a reversed socio-economic gradient [24]. On the other hand, no consistent socioeconomic differences in alcohol consumption have been confirmed among adolescents. The relationship between SEP and alcohol consumption is usually weak [6,24,27,29] or reversed (compared to adult socioeconomic gradients) [26,30]. While binge drinking is associated with lower socioeconomic groups, some studies report that regular but moderate drinking is more common in higher socioeconomic groups [31,32]. Similarly in marijuana use among adolescents, mostly no [6,29] or reversed socioeconomic [29] gradients have been reported among adolescents.

Furthermore, considerable gender differences can be found with relation to health-related behaviour, both in adults and in adolescents. Generally, males exhibit more health-risk and less health-protective behaviour than females [33,34]. However, in recent years some studies have reported a remarkable increase in smoking among women [33-35]. A sedentary lifestyle is also more common for women [13,36-38].

Several studies have shown differences by gender in socioeconomic gradients in HRB. In a sample of Australian adolescents, Scragg et. al.[39] found a negative association between SEP and smoking only among females; the lower was the SEP the higher was the smoking occurrence. Nevertheless, the majority of the studies focusing on gender differences for socioeconomic gradients in smoking found no difference [21]. We have also found no study showing gender differences in the socioeconomic gradient of alcohol and marijuana consumption among adolescents. On the other hand, several adolescent studies report that they only found associations between physical activity and SEP for females [22,40,41]. However, the majority of the studies found no significant differences in the association between SEP and physical activity between boys and girls [21].

Several studies have also gender differences in the relationship between health behaviours and adolescents' self-perceived health. A recently published study on a representative sample of Hungarian adolescents aged 14 to 19 suggests that among boys drug use and the lack of physical activity are significant predictors of self-perceived health, but not among girls. Among girls, smoking may act in a similar way [42]. Gender differences in health perceptions are often reported. This is probably because women, in contrast to men, consider a broader set of factors when making general ratings of health, e.g., psychological factors and minor subjective health complaints [43].

It should be noted that there may be shortcomings in the use of parental SEP markers as measures of social status during adolescence [44]. Traditionally, adolescent studies assess the SEP of the parents (e.g., parental education, parental occupation, family income) as indicators of SEP. As adolescents spend less time at home and experience transition into the independence of adulthood, parental SEP markers may not be accurate indicators of adolescents' social status [21]. It is possible that, during adolescence, HRB is more strongly influenced by peer social status (i.e. the social standing of an adolescent within his/her school), as opposed to family social status. An adolescent's family social status is an assigned status and its impact may be too distant, as teens gain independence, to impact their health behaviour choices [45]. Status among their peers, however, is an earned status, and may better capture the experience of placement within a social hierarchy during adolescence [46].

Most of these studies of socioeconomic differences in HRB in adolescents, however, were cross-sectionally designed. The use of a longitudinal design may be highly significant in exploring changes in HRB, particularly in adolescents. The aim of this study was to analyze the changes in HRB in relation to SEP in a cohort of Slovak adolescents aged between 15 and 19. Our attention is accordingly focused on the following research questions. Did the HRB of adolescents change between the ages 15 and 19? Were the changes in HRB during adolescence related to SEP? If yes, which socioeconomic indicators showed the steepest graduation in HRB?

## Methods

### Sample

The data used in this study were derived from a longitudinal study of socioeconomic inequalities in health [1]. Data for the baseline study (T1) were collected in autumn 1998. The sample consisted of 1850 first-year students from 31 secondary schools located in Kosice, Slovakia. Individual schools and classes were selected randomly after stratification by gender and secondary school type (grammar schools, specialised secondary schools and apprentice schools). The aim of the stratification was to get a similar number of boys and girls and to maintain the proportion of secondary school types similar to the relative share of school types as the overall the national level.

The mean age of the participants at baseline was 14.9, compared to 18.8 at follow up. At baseline respondents completed the questionnaire in their classrooms at school under the guidance of field workers; the response rate was 96.3% [1]. The follow up (T2) took place during December 2002. Respondents received self-administered postal questionnaires along with a stamped return envelope. One reminder was sent to those who did not reply. We received 844 usable questionnaires, representing a response rate of 45.5%. Females were over-represented in the response group as compared with the non-response group. In the response group more grammar students and fewer apprentice students enrolled in the second wave of the study. The potential effect of selective loss to follow-up was assessed by computing Cohen's W effect size for differences in socioeconomic position by response status [47]. All differences were trivial or small (Cohen's W ranging from 0.042 to 0.224), but they were largest for the educational level of the respondents (Table 1). Also differences in HRB in the response group as compared with the non-response at the time of the baseline study were assessed. The differences were trivial in size (Cohen's W ranging from 0.005 to 0.098; Table 2). The potential effect of selective loss according to SEP of respondents is small and according to their HRB even smaller.

**Table 1:** Gender, age and socioeconomic position characteristics ^1^

		Measurement point			
			
		T1 % (N)	T2 drop-out % (N)	T2 participants % (N)	Cohen's **w**^2^
Total		100 (1850)	100 (1006)	100 (844)	
					
Gender	Males	48.6 (899)	53.5 (583)	42.8 (361)	0.107
	Females	51.4 (951)	46.5 (468)	57.2 (483)	
					
Age	Mean (SD)	14.9 (0.62)	18.8 (0.55)	18.8 (0.55)	
					
Respondents' education level	Grammar	23.8 (440)	19.1 (193)	29.3 (247)	0.224
	Specialised secondary	43.4 (802)	41.7 (420)	45.3 (382)	
	Apprentice	32.7 (608)	39.1 (393)	25.5 (215)	
					
Current employment status	Student	n.a.	n.a.	66.3 (558)	n.a.
	Employed	n.a.	n.a.	12.6 (106)	
	Unemployed	n.a.	n.a.	21.1 (178)	
					
Parents' highest occupational level	High	29.8 (538)	29.0 (283)	30.8 (255)	0.052
	Medium	36.2 (653)	34.8 (339)	37.9 (314)	
	Low	33.9 (612)	36.2 (353)	31.3 (259)	
					
Parents' highest education level	High	26.0 (477)	25.7 (255)	26.4 (222)	0.042
	Medium	49.7 (910)	48.3 (479)	51.2 (431)	
	Low	24.3 (445)	25.9 (257)	22.4 (188)	

^1 ^Due to rounding, not all percentages add up to 100%

^2 ^Cohen's w is a measure of the strength of the effect of a characteristic on the outcome. It is independent from sample size, and is expressed as effect size (ES). It could be interpreted as follows: if w < 0.1 the effect is trivial, if w ranges from 0.1 to 0.3 the effect is small, if w ranges from 0.3 to 0.5 the effect is moderate and if w > 0.5 the effect is large.

^3 ^ES – Effect size,

n.a. – not available

T1 – baseline measurement

T2 – follow up

**Table 2:** Health-related behaviour at T1, comparison of participants and drop-outs at T2

		participants at T1 % (N)	drop-out at T2 % (N)	participants at T2 % (N)	Cohen's **w**^1^
Males	Smoking	23.9 (214)	26.1 (140)	20.6 (74)	0.064
	Alcohol	12.8 (115)	12.7 (68)	13.0 (47)	0.005
	Marijuana	7.3 (65)	9.3 (50)	4.2 (15)	0.098
	No sport	9.1 (82)	7.1 (38)	12.2 (44)	0.087

Females	Smoking	18.2(173)	18.6 (87)	17.8 (86)	0.010
	Alcohol	8.3(79)	7.9 (37)	8.7 (42)	0.014
	Marijuana	5.5 (52)	5.2 (25)	5.8 (27)	0.013
	No sport	26.7 (254)	26.5 (128)	26.9 (126)	0.005

1 Cohen's w is a measure of the strength of the effect of a characteristic on the outcome. It is independent from sample size, and is expressed as effect size (ES). It could be interpreted as follows: if w < 0.1 the effect is trivial, if w ranges from 0.1 to 0.3 the effect is small, if w ranges from 0.3 to 0.5 the effect is moderate and if w > 0.5 the effect is large.

T1 – baseline measurement

T2 – follow up

### Indicators of socioeconomic position (SEP)

Four indicators of the adolescents' SEP were used – their current education level and employment status, and the highest education level and highest occupational status of their parents. The respondents' employment status was assessed at follow up; the other socioeconomic indicators were assessed at baseline. *The respondents' education level *was defined as the highest level of education attained. It was classified as – I. Grammar school, II. Specialised secondary school, and III. Apprenticeship or elementary school only. *The respondents' current employment status *was classified as – I. Student, II. Employed and III. Unemployed. *The parents' education level *was based on the parent with the highest level of education attained. It was classified as – I. University, II. Secondary school and III. Apprenticeship or primary school only.

*The parents' occupational status *was based on the parent with the highest occupational status, defined as the parent's current or previous occupation if not currently employed. The occupation was derived by coding job descriptions according to the ISCO88 classifications (International Standard Classification of Occupations). Ten ISCO88 categories were clustered in three groups. High SEP – I. Legislators, II. Senior officials and managers; Medium SEP – III. Technicians and associate professionals, IV. Clerks, and 0. Armed forces (As the professional part of the army consisted mostly of technicians, clerks or managers, we decided to classify the armed forces into the Medium SEP group); Low SEP – V. Service workers and shop and market sales workers, VI. Skilled agricultural and fishery workers, VII. Craftsmen and related trade workers, VIII. Plant and machine operators and assemblers, and IX. Elementary occupations.

### Measures of health-risk behaviour

This study focuses on four types of health-related behaviour – smoking, alcohol use, experience with marijuana and lack of physical exercise. For each, a dichotomised variable was constructed. The main goal of this dichotomization was to analyse possible social inequalities in relation with the presence or absence of the health related behaviours in question. In general, we adhered to cut-offs that had been used in previous studies [1,6,48].

Regarding *smoking habits*, respondents were asked: 'Have you ever smoked a cigarette?' Four possible answers were available – 1) I have never smoked, 2) Yes, I have tried, 3) Sometimes I smoke but not daily, and 4) I smoke daily now. Subjects who smoked sometimes or daily were classified as smokers, the rest as non-smokers.

Regarding *alcohol consumption*, respondents were asked a question concerning their frequency of alcohol consumption over the previous four weeks. Individuals were classified as alcohol consumers if they reported consumption three times or more over the preceding four weeks.

*Experience with marijuana *was assessed by the question: 'Have you ever used marijuana or hash?' Respondents who answered yes were classified as marijuana users, the rest of the respondents as non-users.

Sufficient *physical activity *was assessed by the question: 'How often do you do sport?' There were four possible answers – 1) daily, 2) 2 to 3 times a week, 3) once a week and 4) I do no sport. Only sporting activities lasting longer than 20 minutes were considered and physical education at school was omitted. Respondents were sorted into two groups according to their answers – 1) insufficient physical activity, made up of respondents doing sport once or less a week, and 2) sufficient physical activity, made up of respondents doing sport twice or more a week.

### Statistical analysis

Changes in HRB between the ages of 15 and 19 by SEP category were analyzed using the nonparametric McNemar test for two related dichotomous variables; analyses were stratified by gender and also by type of secondary school.

Formal testing of the interaction of changes in health-related behaviour for gender by SEP was performed. Results showed a statistically significant interaction (p < 0.05) in marijuana use for gender by all socio-economic indicators. We also found statistically significant interactions with gender (p < 0.05) of alcohol consumption and of smoking for all socio-economic indicators except for the respondents' current employment status. With regard to physical activity, statistically significant interactions (p < 0.05) with gender were found for the parents' education level and for the parents' occupational level. Because of this, we present all results for males and females to support comparisons of socioeconomic gradients across various HRBs.

Changes in HRB gradients with regard to SEP were analyzed using logistic regression. For each type of HRB, three regression models were explored – Model 1 to examine the effect of SEP on HRB at T1; Model 2 to examine the effect of SEP on HRB at T2; and Model 3 to examine the potential differences in changes in socioeconomic gradients in HRB between 15 and 19 by analyzing the effect of SEP on HRB at T2 controlled for HRB at T1. The procedure was repeated for all four socioeconomic indicators used.

All analyses were carried out separately for males and females. The analyses were all done using the statistical software package SPSS version 10.1. Using MlWin 2.02,[49] we found no indications for a clustering by school at the baseline measurement for the outcomes at the follow-up.

## Results

### Changes in HRB between age 15 (T1) and 19 (T2)

As the results of the McNemar tests show, alcohol consumption, experience with marijuana and insufficient physical activity in males at T2 compared to T1 statistically significantly increased for each category of each socioeconomic indicator. The same applies to smoking behaviour, with the exception of males at the lowest education level, unemployed males and males with parents at the lowest education level who did not report statistically significant worsening in smoking behaviour (Table 3).

**Table 3:** Socioeconomic gradients in health-risk behaviour of males

**Smoking**		T1%	T2%	Sig.^1^	model 1 OR	95 % CI	p	model 2 OR	95 % CI	p	model 3 OR	95 % CI	p
Respondents' education level	*Grammar*	3.0	23.8	0.000	**1**		^2^	**1**		^2^	1		
	*Specialised*	18.3	36.4	0.000	**7.32**	(2.16–24.7)		**1.85**	(1.04–3.20)		1.27	(0.69–2.31)	
	*Apprentice*	40.6	50.0	0.064	**22.30**	(6.63–74.9)		**3.21**	(1.77–5.82)		1.34	(0.68–2.66)	
Current employment status	*Student*	13.7	31.1	0.000	**1**		^2^	**1**		^2^	1		
	*Employed*	31.3	59.4	0.004	**2.87**	(1.26–6.55)		**3.23**	(1.53–6.88)		2.65	(1.15–6.13)	
	*Unemployed*	43.8	50.0	0.481	**4.90**	(2.66–8.99)		**2.21**	(1.27–3.87)		1.11	(0.56–2.18)	
Parents' highest education level	*High*	12.7	28.9	0.001	**1**		^2^	**1**		^2^	1		
	*Medium*	21.2	39.0	0.000	**1.84**	(0.93–3.64)		**1.78**	(1.04–2.98)		1.57	(0.87–2.82)	
	*Low*	28.8	41.2	0.064	**2.77**	(1.28–5.98)		**1.84**	(0.96–3.49)		1.30	(0.63–2.71)	
Parents' highest occupational level	*High*	12.4	27.5	0.000	**1**		^2^	1			1		
	*Medium*	23.0	40.0	0.000	**2.11**	(1.07–4.13)		1.59	(0.93–2.65)		1.27	(0.71–2.29)	
	*Low*	24.7	41.1	0.003	**2.32**	(1.14–4.73)		1.72	(0.98–3.03)		1.34	(0.71–2.54)	

**Alcohol consumption**		T1%	T2%	Sig.^1^	model 1 OR	95 % CI		model 2 OR	95 % CI		model 3 OR	95 % CI	

Respondents' education level	*Grammar*	11.9	26.7	0.000	1			1			1		
	*Specialised*	14.3	33.8	0.000	1.24	(0.58–2.63)		1.40	(0.80–2.43)		1.37	(0.78–2.41)	
	*Apprentice*	12.3	37.7	0.000	1.04	(0.44–2.39)		1.66	(0.92–3.00)		1.68	(0.51–5.58)	
Current employment status	*Student*	12.9	29.9	0.000	1			1			1		
	*Employed*	6.3	46.9	0.000	0.45	(0.10–1.97)		2.07	(0.98–4.34)		2.25	(0.99–4.77)	
	*Unemployed*	17.2	39.1	0.000	1.40	(0.67–2.95)		1.50	(0.85–2.65)		1.45	(0.82–2.58)	
Parents highest education level	*High*	8.8	32.2	0.000	1			1			1		
	*Medium*	14.6	31.6	0.000	1.77	(0.80–3.92)		1.00	(0.60–1.68)		0.94	(0.56–1.59)	
	*Low*	15.1	34.0	0.004	1.83	(0.72–4.68)		1.09	(0.58–2.06)		1.02	(0.53–1.95)	
Parents highest occupational level	*High*	9.9	32.4	0.000	1			1			1		
	*Medium*	16.2	32.4	0.002	1.75	(0.82–3.71)		0.97	(0.58–1.64)		0.90	(0.53–1.54)	
	*Low*	9.3	34.2	0.000	0.93	(0.37–2.31)		1.08	(0.62–1.91)		1.09	(0.62–1.94)	

**Marijuana use**		T1%	T2%	Sig.^1^	model 1 OR	95 % CI		model 2 OR	95 % CI		model 3 OR	95 % CI	

Respondents' education level	*Grammar*	1.0	25.7	0.009	**1**		^2^	**1**		^2^	1		
	*Specialised*	2.6	39.6	0.000	**2.67**	(0.29–24.2)		**1.89**	(1.09–3.28)		1.84	(1.05–3.22)	
	*Apprentice*	9.4	42.9	0.000	**10.42**	(1.31–82.9)		**2.16**	(1.20–3.90)		1.79	(0.97–3.29)	
Current employment status	*Student*	3.8	33.0	0.000	1			**1**		^2^	1		
	*Employed*	3.1	43.8	0.000	0.82	(0.99–4.77)		**1.58**	(0.75–3.33)		1.65	(0.77–3.52)	
	*Unemployed*	6.3	49.2	0.000	1.69	(0.51–5.58)		**1.97**	(1.13–3.44)		1.93	(1.00–3.43)	
Parents highest education level	*High*	2.0	30.6	0.000	1			1			1		
	*Medium*	5.4	41.2	0.000	2.86	(0.61–13.3)		1.53	(0.91–2.55)		1.42	(0.84–2.41)	
	*Low*	2.7	35.4	0.000	1.41	(0.19–10.2)		1.30	(0.68–2.45)		1.28	(0.66–2.45)	
Parents highest occupational level	*High*	2.5	30.4	0.000	1			1			1		
	*Medium*	5.1	40.0	0.000	2.13	(0.54–8.44)		1.59	(0.95–2.66)		1.52	(0.89–2.58)	
	*Low*	3.1	36.1	0.000	1.26	(0.25–6.36)		1.25	(0.70–2.20)		1.24	(0.69–2.21)	

**Insufficient physical activity**		T1%	T2%	Sig.^1^	model 1 OR	95 % CI		model 2 OR	95 % CI		model 3 OR	95 % CI	

Respondents' education level	*Grammar*	5.0	21.8	0.001	**1**		^2^	1			1		
	*Specialised*	15.6	31.2	0.000	**3.55**	(1.31–9.63)		1.63	(0.91–2.91)		1.39	(0.76–2.53)	
	*Apprentice*	14.2	29.5	0.006	**3.17**	(1.11–9.06)		1.50	(0.80–2.83)		1.31	(0.68–2.50)	
Current employment status	*Student*	11.4	27.0	0.000	1			1			1		
	*Employed*	15.6	34.4	0.046	1.44	(0.52–4.03)		1.42	(0.65–3.09)		1.35	(0.60–3.02)	
	*Unemployed*	14.1	29.7	0.031	1.28	(0.57–2.84)		1.14	(0.63–2.08)		1.10	(0.59–2.05)	
Parents' highest education level	*High*	10.8	27.3	0.002	1			1			1		
	*Medium*	11.4	30.9	0.000	1.06	(0.49–2.30)		1.18	(0.69–2.04)		1.18	(0.68–2.06)	
	*Low*	15.1	22.9	0.006	1.47	(0.60–3.60)		0.91	(0.46–1.81)		0.84	(0.41–1.71)	
Parents' highest occupational level	*High*	9.9	26.5	0.000	1			1			1		
	*Medium*	14.0	29.9	0.000	1.48	(0.68–3.18)		1.19	(0.69–2.05)		1.13	(0.65–1.96)	
	*Low*	11.3	24.7	0.021	1.16	(0.49–2.76)		0.79	(0.43–1.47)		0.76	(0.40–1.45)	

1) McNemar test

2) text in bold indicate that overall a variable contributes to the logistic model at p < 0.05

*Model 1 *effect of SEP on HRB at T1; *Model 2 *effect of SEP on HRB at T2; *Model 3 *effect of SEP on HRB at T2 controlled for HRB at T1

OR – odds ratio

Among females, a statistically significant increase in smoking, experience with marijuana and insufficient physical activity was reported for each category of each socioeconomic indicator. The same applies to alcohol consumption with the exception of females at the lowest education level, unemployed females and females with parents in the lowest educational and occupational levels who did not report statistically significant increases in alcohol consumption (Table 4).

**Table 4:** Socioeconomic gradients in health-risk behaviour of females

**Smoking**		T1%	T2%	Sig.^1^	model 1 OR	95 % CI	p	model 2 OR	95 % CI	p	model 3 OR	95 % CI	p
Respondents' education level	*Grammar*	14.4	38.4	0.000	1			1			1		
	*Specialised*	17.5	40.5	0.000	1.27	(0.71–2.25)		1.10	(0.71–1.68)		1.04	(0.66–1.63)	
	*Apprentice*	22.9	45.9	0.000	1.77	(0.93–3.37)		1.36	(0.82–2.25)		1.19	(0.70–2.03)	
Current employment status	*Student*	14.3	35.4	0.000	**1**		^2^	**1**		^2^	**1**		^2^
	*Employed*	18.9	50.7	0.000	**1.40**	(0.72–2.73)		**1.88**	(1.19–3.15)		**1.84**	(1.06–3.17)	
	*Unemployed*	26.3	50.0	0.000	**2.14**	(1.26–3.64)		**1.83**	(1.18–2.83)		**1.55**	(0.97–2.48)	
Parents' highest education level	*High*	18.3	48.1	0.000	1			**1**		^2^	**1**		^2^
	*Medium*	18.7	38.8	0.000	1.03	(0.58–1.80)		**0.65**	(0.42–1.01)		**0.61**	(0.38–0.98)	
	*Low*	14.8	37.0	0.000	0.77	(0.39–1.54)		**0.59**	(0.34–0.99)		**0.59**	(0.34–1.03)	
Parents' highest occupational level	*High*	20.1	49.6	0.000	1			1			1		
	*Medium*	17.4	39.0	0.000	0.84	(0.47–1.48)		0.68	(0.43–1.08)		0.69	(0.42–1.12)	
	*Low*	16.7	36.5	0.000	0.79	(0.44–1.43)		0.63	(0.40–1.01)		0.64	(0.39–1.06)	

**Alcohol consumption**		T1%	T2%	Sig.^1^	model 1 OR	95 % CI		model 2 OR	95 % CI		model 3 OR	95 % CI	

Respondents' education level	*Grammar*	9.6	24.7	0.001	1			**1**		^2^	**1**		^2^
	*Specialised*	9.2	18.9	0.002	0.96	(0.47–1.95)		**0.71**	(0.43–1.17)		**0.72**	(0.43–1.19)	
	*Apprentice*	6.4	11.0	0.332	0.65	(0.25–1.66)		**0.38**	(0.19–0.77)		**0.39**	(0.19–0.78)	
Current employment status	*Student*	10.2	19.1	0.002	1			1			1		
	*Employed*	2.7	21.6	0.001	0.24	(0.06–1.05)		1.17	(0.63–2.18)		1.24	(0.66–2.32)	
	*Unemployed*	8.8	16.7	0.078	0.85	(0.40–1.79)		0.85	(0.48–1.50)		0.85	(0.48–1.52)	
Parents highest education level	*High*	14.2	25.4	0.021	1			**1**	^2^		**1**		^2^
	*Medium*	7.3	19.2	0.001	0.48	(0.23–0.97)		**0.59**	(0.35–0.99)		**0.61**	(0.36–1.03)	
	*Low*	6.1	13.6	0.096	0.39	(0.16–1.01)		**0.41**	(0.21–0.81)		**0.43**	(0.22–0.85)	
Parents highest occupational level	*High*	14.9	26.7	0.040	**1**		^2^	**1**		^2^	1		
	*Medium*	5.6	17.6	0.000	**0.34**	(0.15–0.75)		**0.70**	(0.41–1.20)		0.74	(0.43–1.28)	
	*Low*	7.4	13.0	0.089	**0.46**	(0.21–0.97)		**0.46**	(0.25–0.84)		0.48	(0.27–0.88)	

**Marijuana use**		T1%	T2%	Sig.^1^	model 1 OR	95 % CI		model 2 OR	95 % CI		model 3 OR	95 % CI	

Respondents' education level	*Grammar*	4.8	32.2	0.000	1			1			1		
	*Specialised*	4.8	28.2	0.000	1.01	(0.38–2.66)		0.83	(0.53–1.30)		0.82	(0.51–1.30)	
	*Apprentice*	6.5	22.0	0.000	1.38	(0.47–4.04)		0.60	(0.34–1.05)		0.55	(0.30–1.04)	
Current employment status	*Student*	5.1	29.3	0.000	1			1			1		
	*Employed*	1.4	27.4	0.000	0.26	(0.33–1.96)		0.91	(0.52–1.62)		1.00	(0.56–1.79)	
	*Unemployed*	8.0	25.4	0.000	1.61	(0.68–3.79)		0.84	(0.51–1.35)		0.76	(0.45–1.27)	
Parents highest education level	*High*	5.8	38.1	0.000	1			**1**		^2^	**1**		
	*Medium*	4.5	24.2	0.000	0.76	(0.29–2.00)		**0.51**	(0.32–0.81)		**0.50**	(0.31–0.81)	
	*Low*	6.1	23.6	0.000	1.06	(0.36–3.11)		**0.42**	(0.24–0.74)		**0.39**	(0.21–0.71)	
Parents highest occupational level	*High*	6.7	40.0	0.000	1			**1**		^2^	**1**		^2^
	*Medium*	5.1	25.3	0.000	0.74	(0.29–1.92)		**0.52**	(0.32–0.85)		**0.52**	(0.31–0.86)	
	*Low*	4.3	21.7	0.000	0.63	(0.23–1.73)		**0.50**	(0.30–0.83)		**0.51**	(0.30–0.86)	

**Insufficient physical activity**		T1%	T2%	Sig.^1^	model 1 OR	95 % CI		model 2 OR	95 % CI		model 3 OR	95 % CI	

Respondents' education level	*Grammar*	25.3	36.3	0.038	1			**1**		^2^	**1**		^2^
	*Specialised*	23.7	42.1	0.000	0.91	(0.57–1.48)		**1.28**	(0.83–1.96)		**1.32**	(0.85–2.05)	
	*Apprentice*	33.9	56.0	0.001	1.51	(0.88–2.61)		**2.23**	(1.34–3.70)		**2.13**	(1.26–3.58)	
Current employment status	*Student*	23.8	38.8	0.000	1			**1**			1		
	*Employed*	28.4	50.0	0.006	1.27	(0.72–2.25)		**1.58**	(0.95–2.64)	^2^	1.54	(0.91–2.61)	
	*Unemployed*	31.6	51.8	0.001	1.48	(0.92–2.38)		**1.69**	(1.10–2.62)		1.60	(1.02–2.51)	
Parents' highest education level	*High*	24.2	36.6	0.067	1			**1**		^2^	**1**		^2^
	*Medium*	27.2	43.8	0.000	1.18	(0.71–1.94)		**1.51**	(0.96–2.37)		**1.49**	(0.93–2.38)	
	*Low*	26.1	50.0	0.000	1.11	(0.61–2.00)		**2.10**	(1.24–3.56)		**2.15**	(1.25–4.71)	
Parents' highest occupational level	*High*	24.6	34.2	0.021	1			**1**		^2^	**1**		^2^
	*Medium*	30.9	43.9	0.008	1.37	(0.83–2.27)		**1.35**	(0.85–2.43)		**1.28**	(0.80–2.06)	
	*Low*	22.8	52.2	0.000	0.91	(0.53–1.55)		**1.74**	(1.09–2.77)		**1.83**	(1.13–2.97)	

1) McNemar test

2) text in bold indicate that overall a variable contributes to the logistic model at p < 0.05

*Model 1 *effect of SEP on HRB at T1

*Model 2 *effect of SEP on HRB at T2

*Model 3 *effect of SEP on HRB at T2 controlled for HRB at T1

OR – odds ratio

### Types of socioeconomic gradients reported

Two types of socioeconomic gradients were found. The first type was the "traditional" (consistent with adult literature) socioeconomic gradient, characterised by a decreasing prevalence of unhealthy behaviour associated with increasing SEP. This means the higher the respondent's SEP the lower the level of unhealthy behaviour. The second was a "reversed" socioeconomic gradient characterised by an increasing prevalence of unhealthy behaviour with increasing SEP. This means the higher the respondent's SEP the higher the level of unhealthy behaviour.

### Changes in socioeconomic gradients in smoking

The results of the logistic regression indicate that the SEP of males is a statistically significant predictor of smoking behaviour at T1 and at T2. Clear traditional socioeconomic gradients in smoking at T1, less smoking associated with higher SEP, were found for all four socioeconomic indicators used. Similarly, statistically significant social gradients were found at T2 according to the education levels of respondents, their current employment status and the highest education level of their parents. The statistically significant effect of SEP on smoking behaviour at T2 disappeared when controlled for smoking behaviour at T1 (Table 3).

A less clear picture was found of changes in the smoking habits of females. A traditional gradient in smoking among females according to current employment status was found for both measurement points. The gradient at T2 remained statistically significant even after being controlled for smoking behaviour at T1. However, a statistically significant reversed gradient in smoking according to the highest education level of the parents was found at T2. Moreover, this gradient at T2 remained statistically significant even after being controlled for smoking behaviour at T1 (Table 4).

### Changes in socioeconomic gradients in alcohol consumption

No socioeconomic differences in alcohol consumption among males were found at T1 or at T2 (Table 3). On the other hand, among females, reversed socioeconomic gradients were again found – at T1 according to the highest occupational level of the parents and at T2 according to the respondents' education level and the highest educational and occupational levels of the parents. After controlling gradients at T2 for alcohol consumption at T1, the gradients according to the respondents' education level and highest education level of their parents remained statistically significant, while the gradient according to highest occupational level of the parents became statistically insignificant (Table 4).

### Changes in socioeconomic gradients in experience with marijuana

A traditional gradient according to the respondents' education level in experience with marijuana was found among males at both times of measurement. A traditional gradient according to the respondents' current employment status was also found but only at T2. After controlling for experience with marijuana at T1, gradients at T2 became statistically insignificant (Table 3).

Again, a different picture was found for females. No socioeconomic differences in the experience with marijuana were found in relation to the respondents' employment status and also to their education level at both measurement points. On the other hand, clear reversed gradients according to the highest educational and occupational levels of the parents were found. These remained stable even after controlling for experience with marijuana at T1 (Table 4).

### Changes in socioeconomic gradients in insufficient physical activity

Regarding the insufficient physical activity of males at T1, only a traditional socioeconomic gradient according to the respondents' education level was found. No statistically significant socioeconomic differences among males were found at T2 (Table 3).

On the other hand, obvious socioeconomic gradients regarding changes in insufficient physical activity were found for females. Since at T1 no socioeconomic gradients according any of the socioeconomic indicators used were found, at T2, clear traditional socioeconomic gradients were found for every socioeconomic indicator used. socioeconomic gradients according to the respondents' education level and the highest educational and occupational levels of their parents remained statistically significant even after being controlled for insufficient physical activity at T1 (Table 4).

### Gender differences

The relative increase in the occurrence of risky behaviour in alcohol consumption, experience with marijuana and insufficient physical activity between T1 and T2 was greater in males than in females. These gender differences were statistically significant (Table 5). No gender differences were found in the change in smoking behaviour.

**Table 5:** Differences in changes of HRB in the period between T1 and T2 according to gender (results of logistic regression)

			Crude			Adjusted for HRB at T1		
	T1(%)	T2(%)	OR	95% CI	p value	OR	95% CI	p value
**Smoking**								
Males	20.6	36.8	1			1		
Females	17.8	41.1	1.20	(0.90, 1.20)	0.213	1.32	(0.97, 1.80)	0.078
**Alcohol**								
Males	13.0	33.0	1			1		
Females	8.7	18.9	0.47	(0.34, 0.47)	***0.000***	0.49	(0.36, 0.67)	***0.000***
**Marijuana**								
Males	4.2	36.7	1			1		
Females	5.2	28.0	0.40	(0.28, 0.40)	***0.000***	0.64	(0.47, 0.87)	***0.004***
**No sport**								
Males	12.2	18.1	1			1		
Females	26.5	43.5	0.67	(0.50, 0.67)	***0.008***	1.70	(1.25, 2.30)	***0.001***

OR – odds ratio

T1 – baseline measurement

T2 – follow up measurement

95% CI – confidence interval

## Discussion

This study describes changes in HRB according to SEP in a cohort of Slovak young adults aged between 15 and 19. Between these ages a greater increase in alcohol consumption and experience with marijuana was found for males than in females. However, the increase in insufficient physical activity was greater for females. Since there was already a clear gender difference at the study baseline, the gap between the genders in relation to these HRBs became wider. The finding that more males drank alcohol and used marijuana was unsurprising as similar outputs had been published in earlier studies [13,37,50-53]. A sedentary lifestyle is more common among females than in males in late adolescence and young adulthood [13,37,52,53]. High consumption of alcohol is likely to be linked to the young males' lifestyle, associated with a normative peer pressure to drink [34]. The reasons for marijuana use among men may be similar to those for alcohol, and peer attitudes play an important role in explaining this [54].

Studies performed over the last few decades present a well-documented equalisation trend in the smoking behaviour of males and females. This has resulted in an increasing number of smoking females in the community [34,35], while the proportion of smoking males is decreasing [35] or remaining stable [11].

As no socioeconomic differences in changes in the HRB studied were found among males, the socioeconomic gradients in HRB described at T1 were similar to those at T2 for males. The number of smokers was highest in the lowest socioeconomic groups for all socioeconomic indicators. A similar outcome was obtained when using marijuana and insufficient sporting activity among males, but not for every socioeconomic indicator. No socioeconomic gradient was found for alcohol consumption by males. These findings are consistent with the results of previous studies [32,55,56].

Results on experience with marijuana are somewhat difficult to understand. While in relation to women's educational level and to their employment status no socioeconomic gradient was found, in relation to their parental educational and occupational level a reversed gradient was discovered. On the other hand, among men traditional gradients were found in relation to their education level and to their employment status, and no gradients in relation to parental socioeconomic indicators. However, this ambiguous result fits with those of previous studies on the association of SEP and marijuana experience during adolescence. According to the current literature review by Hanson and Chen [21], these studies also found varying associations. The most frequently reported finding was of no socioeconomic gradients, while some reported traditional socioeconomic gradients: higher SEP associated with less use; and some reported reversed socioeconomic gradients: the higher the SEP, the greater the use. The character of the socioeconomic gradients was usually determined by the type of socioeconomic indicator used. The findings of previous studies suggest that the relationship between the social status of parents (e.g. educational or occupational status) as socioeconomic indicators and marijuana use is more likely to show no [29,57] or a traditional association, [58,59] whereas the relationship between financial resources [60] or self-assessed SEP [29] as indicators and marijuana use is more likely to be reversed. However, there are studies with results not fully consistent with these findings [21].

According to Luthar and Latendresse, [61] high-SEP adolescents engage in negative health behaviours in order to combat the stress, anxiety, and depression they experience from achievement pressures. It is possible that this type of pressure could be common in relation to highly-educated parents with a less academically successful child. In combination with more available money and negative peer influence, the group having parents with high social status could become more susceptible to negative health-related behaviour. These explanations require more attention in future.

Using SEP based on the parents' characteristics yielded an inconsistent or reversed pattern of socioeconomic differences in smoking among females, while using SEP based on the adolescents themselves – their current position – yielded socioeconomic differences in smoking unfavourable for females of lower SEP. An explanation may be that measuring SEP using the parents' characteristics loses validity in adolescence [3,7]. On the other hand, using the socioeconomic characteristics of adolescents is also problematic [4]. Methodological problems related to measuring SEP may be a source of inconsistency in findings related to socioeconomic differences in HRB. Glendinning et al. [62], with the aim of measuring SEP based on respondents' instead of their parents' characteristics, used young adults' economic activity. It is suggested that subjects who continue to study will differ as regards SEP from those who enter the labour market and succeed (employed) or fail (unemployed). Hanson and Chen [21] conclude in a current literature review of socioeconomic position and health related behaviour in adolescence that future studies should employ alternative measures of SEP, such as an adolescent's perception of social status relative to others in their peer group, due to recent findings which suggest that such measures may be a better predictor of adolescent health than the traditional objective measures. By employing alternative measures of social status, future studies may be able to further clarify the socioeconomic patterns for adolescent health behaviours.

Findings related to socioeconomic differences in HRB may depend on the way HRB is measured. Sweeting and West explored socioeconomic differences in smoking among adolescents with respect to the definition of smokers. The stricter the definition of smoking, the clearer the socioeconomic gradients in smoking that were found. Moreover, reversed relationships between SEP and HRB were found when occasional smoking was explored [5]. Our definition of smokers includes both daily and occasional smokers. We therefore repeated post-hoc our analyses for daily smokers. The character of socioeconomic gradients in smoking among males remained stable. On the other hand, results for females were more in line with findings of Sweeting and West; the traditional gradients previously found according to their employment status and according to their education level became steeper and the reversed socioeconomic gradient previously found according to their parents' educational level was no more statistically significant.

Changes in alcohol consumption in females were mostly related to the education levels of their parents and the respondents' own education levels. Females with higher SEP reported greater increase in alcohol consumption compared to females with lower SEP. Moderate drinking may be part of a high SEP lifestyle geared towards pleasure and comfort [31,32,55,56].

No statistically significant socioeconomic differences among males were found at T2. On the other hand, obvious socioeconomic gradients regarding insufficient physical activity were found for females at T2. This is consistent with the results of several previous studies [22,40,41]. Female respondents' school type and the education level of their parents appeared to be statistically significant predictors of physical inactivity. The largest increase in insufficient physical activity was observed among females with low education and with low-educated parents. Females may be more likely to exercise if they are enrolled in a formal activity, such as a dance class or swimming lessons [63]. Even though lower education is usually associated with lower income, the inaccessibility of sports facilities due to high costs could explain this effect only partially. Overall, these findings suggest that higher family social status or prestige may be a stronger influence on physical activity than financial resources in high SEP adolescents [21]. The explanation for this relationship is more likely to be a kind of normative behaviour towards damaging behaviours [64] and better ability to make informed choices [14,63].

We have not adjusted the analysis of socioeconomic gradients in one health-related behaviour with regard to the other health-related behaviours. Health-related behaviours may indeed cluster, but it is open to discussion whether they should be considered as confounders of each other, i.e. having a causal relationship with both the determinant (change of age) and the outcome under study, i.e. another health-related behaviour. We think that the most valid approach is to consider them as outcomes with partially identical causes (such as SEP), but having no direct causal effects on each other. For that reason, we have not adjusted our analyses for the effects of other outcomes.

This study has several strengths and limitations. A major strength of our study is its longitudinal design. The main limitation of this study, just as in every longitudinal research project using self-administered postal questionnaires in adolescents, is the relatively low response rate. Compared to females and better-educated males, low-educated males responded slightly less. However, differences in response rates by SEP were relatively small, thereby biased results due selective non-response are less probable. The period of young adulthood is associated with changes of permanent residence. Some of the respondents became independent of their parents and changed residence; large numbers of respondents became simply unreachable because of study or work in another part of the country or abroad. According to data from the Statistical Office of Slovak Republic, about 7.5% of all employed Slovak citizens were employed abroad. A very substantial part of this group consists of young people aged from 18 to 30 years. This may explain some of the differences in response rate we found in terms of gender and SEP, but probably only a part of them. Another limitation stems from the way in which we performed the McNemar test. Data was stratified not only by gender but also by every indicator of SEP and this resulted in 48 different p-values. Multiple testing may have affected some of our findings.

## Conclusion

In conclusion, our follow-up study contributes to the debate on health inequality by investigating the relationship between SEP and health-related behaviour in late adolescence and early adulthood, which is relatively rarely investigated. The results show that the dynamics of HRB change are related to SEP and gender. Socioeconomic differences in HRB established in adolescence remained stable until young adulthood among males but not among females. Some diminution of the gender gap in smoking was confirmed.

Initiation into HRB takes place during the turmoil of adolescence, which is characterized by many personal and social changes. It is difficult to capture and understand the dynamics within which the uptake of HRB takes place. More longitudinal research is needed to fully understand the process by which age, SEP and HRB influence health.

## Competing interests

The author(s) declare that they have no competing interests.

## Authors' contributions

FS carried out the data collection, did the analysis, interpreted the data, and drafted the manuscript. JPvD participated in the design of the study and helped to draft the manuscript. AMG participated in the design and the coordination of the study and helped to draft the manuscript. MS participated in the data collection, participated in the coordination and helped to draft the manuscript. JWG participated in the design of the study and helped to draft the manuscript. SAR commented on the design of the study, contributed to the statistical analysis and helped to draft the manuscript. All authors read and approved the final manuscript.

## Pre-publication history

The pre-publication history for this paper can be accessed here:


